# Ambulatory assessed implicit affect is associated with salivary cortisol

**DOI:** 10.3389/fpsyg.2015.00111

**Published:** 2015-02-10

**Authors:** Joram C. L. Mossink, Bart Verkuil, Andreas M. Burger, Marieke S. Tollenaar, Jos F. Brosschot

**Affiliations:** ^1^Clinical Psychology, Faculty of Social Sciences, Leiden University, LeidenNetherlands; ^2^Health Psychology, Faculty of Social Sciences, Leiden University, LeidenNetherlands

**Keywords:** unconscious stress, implicit affect, sadness, cortisol, daily life, IPANAT, cortisol awakening response

## Abstract

One of the presumed pathways linking negative emotions to adverse somatic health is an overactive HPA-axis, usually indicated by elevated cortisol levels. Traditionally, research has focused on consciously reported negative emotions. Yet, given that the majority of information processing occurs without conscious awareness, stress physiology might also be influenced by affective processes that people are not aware of. In a 24-h ambulatory study we examined whether cortisol levels were associated with two implicit measures. Implicit affect was assessed using the Implicit Positive and Negative Affect Test, and implicit negative memory bias was assessed with the word fragment completion tasks. In 55 healthy participants, we measured subjective stress levels, worries, implicit, and explicit affect each hour during waking hours. Also, saliva samples were collected at three fixed times during the day, as well as upon waking and 30 min thereafter (cortisol awakening response). Multilevel analyses of the daytime cortisol levels revealed that the presence of an implicit negative memory bias was associated with increased cortisol levels. Additionally, implicit PA and, unexpectedly, implicit NA were negatively associated with cortisol levels. Finally, participants demonstrating higher levels of implicit sadness during the first measurement day, had a stronger cortisol rise upon awakening at the next day. Contrary to previous research, no associations between explicit affect and cortisol were apparent. The current study was the first to examine the concurrent relation between implicit measures and stress physiology in daily life. The results suggest that the traditional focus on consciously reported feelings and emotions is limited, and that implicit measures can add to our understanding of how stress and emotions contribute to daily physiological activity and, in the long term, health problems.

## INTRODUCTION

In our daily lives we are constantly facing many forms of stress, including social demands, time constraints, and pressure to achieve (e.g., work, family, friends, and deadlines). Yet, stress is so ingrained into our daily lives that we may not always be consciously aware of and/or be able to accurately report that we have elevated stress levels until we feel a sense of relief when we’re not stressed anymore. This inability to perceive and report stress has been termed as ‘unconscious stress,’ which can be defined as the cognitive representation of stressful events when attention is directed elsewhere (Brosschot et al., 2010). While we can easily relate to the notion of unconscious stress, few studies have attempted to study it.

Importantly, chronic stress has serious health consequences as it causes wear and tear on the body and it has been linked to psychological and physiological problems such as chronic fatigue, cardiovascular disease, depression, abdominal obesity as well as specific neuronal changes in the prefrontal cortex, hippocampus, and amygdala (Sapolsky, 1996; Smyth et al., 1998; Rosengren et al., 2004; McEwen, 2005). One of the physiological factors contributing to this wear and tear of the human body is prolonged hyperactivity of the HPA-axis, as indicated by high ultradian levels of cortisol (Lupien et al., 2009). Prolonged enhanced levels of cortisol have been shown to be a risk factor for cardiovascular disease (Manenschijn et al., 2013). Furthermore, a bulk of research is available that reports the associations between a wide variety of stressors and elevated cortisol levels. Documented stressors associated with elevated cortisol levels vary in intensity such as trauma (e.g., Howard et al., 1955; Resnick et al., 1995) daily hassles (e.g., Sher, 2004), life events (e.g., van Eck et al., 1996) as well as stressors that only occur ‘in one’s head,’ such as those worried about (e.g., Schlotz et al., 2004; Zoccola et al., 2008 – for a review see: Verkuil et al., 2010). In addition, in several studies cortisol has been associated with one’s affective state (e.g., Smyth et al., 1998; Jacobs et al., 2007; Chida and Steptoe, 2009). Given that frequently experienced but relatively minor stressors may even lead to elevated cortisol levels, it can be suggested that our body may also respond to stressors that we are not consciously aware of but are nonetheless cognitively represented in the brain. However, only little research has been directed at this topic.

Whereas triggering the physiological stress response through worrying or explicit affect takes place in the absence of an impending stressor, it still involves our conscious attention (Brosschot et al., 2006). Yet, it is believed that most cognitive processes take place rather implicitly, that is, while we are not consciously aware of this (e.g., Bargh and Morsella, 2008), and emotional cognitive processes are not unlikely to be an exception (Winkielman and Berridge, 2004; Brosschot et al., 2010). Brosschot et al. (2010) suggest that stress may affect us implicitly through cognitive representations of stressful events that people are not aware of, that is, unconscious stress. Preliminary – indirect – evidence for this idea comes from studies showing that stress and worries predict cardiovascular activity during sleep (while we are not consciously worrying; Brosschot et al., 2007; Yoshino and Matsuoka, 2009). Furthermore, an ambulatory study showed that after a worry episode has ended, cardiovascular activity can remain high for up to 2 h – a prolonged effect of worry itself that could not be explained by new worry episodes, mood, and lifestyle factors (Pieper et al., 2010). In addition, several experimental studies showed that subliminal negative emotional primes may increase cardiovascular activity (Levy et al., 2000; Hull et al., 2002), and this strongly suggests unconscious NA (stress) to be a mediator. However, thus far, evidence directly linking unconscious stress to physiological activity is scarce.

The fact that unconscious stress’ physiological effects have been under-investigated could have to do with the scarcity of available measures of unconscious affect. One of the measures of implicit affect that has been used for decades is the word fragment completion task, which assesses the extent to which affective information is accessible from memory (e.g., Denny and Hunt, 1992; Anderson et al., 2003). When people complete word fragments in a negative way – i.e., “p – – son” can become “poison” or “person” – this indicates an enhanced accessibility of negative information (implicit negative memory bias), and potentially captures the extent to which people are currently experiencing unconscious stress. However, it is unknown whether implicit negative memory biases are associated with stress-related physiology. We here examined for the first time whether implicit negative memory bias – as assessed with the word fragment completion task – is associated with enhanced cortisol levels assessed at several moments during the day.

Additionally, a new instrument has recently been developed and validated that may make the assessment of unconscious stress possible via the indirect measurement of implicit affect. The Implicit Positive and Negative Affect Test (IPANAT), developed by Quirin et al. (2009a), uses people’s spontaneous emotional judgments on adjectives paired with pseudo-words to assess levels of both positive (PA) and negative (NA) implicit affect. The IPANAT is a test of ‘automatic activation of cognitive representations of affective experiences’ (Quirin et al., 2009a, p. 501), which is very close to the definition of unconscious stress provided above. Importantly, the implicit NA subscale of the IPANAT was found to predict the cortisol response when subjects were confronted with acute stress (Quirin et al., 2009b). Moreover, a parallel study showed that the IPANAT scores for implicit PA are associated with cortisol measures in daily life, i.e., a reduced total circadian cortisol release, specifically due to the association of implicit PA with reduced morning levels of cortisol (Quirin et al., 2009b). In both these studies, no associations were found with explicit PA and NA, suggesting that implicit measures can have independent effects, and thus can really add to our understanding of how and when the physiological stress response is triggered.

In the present study, we aimed to replicate and extend these findings with the IPANAT. Quirin et al. (2009b) only administered the IPANAT once, and thus did not examine whether momentary state fluctuations in implicit NA and PA were associated with momentary fluctuations in cortisol levels. Their results suggests that at least *trait* variance in implicit affect is associated with daily cortisol levels. It is the main aim of the current study to assess whether *state* variance in implicit affect is associated with *state* (momentary) cortisol levels. Specifically, assessing ultradian cortisol secretion, we hypothesized that momentary implicit PA would show a negative association with ultradian cortisol levels, and inversely, that momentary implicit NA is positively associated with ultradian cortisol levels. We examined this association for fixed assessment moments during the day, while controlling for explicit affect and bio-behavioral variables that may influence cortisol levels.

In addition to examining ultradian cortisol secretion, we tested the relationship between implicit affect and the cortisol awakening response (CAR). The CAR is defined as the increase in cortisol secretion that is observed within the first hour after awakening (Chida and Steptoe, 2009). The magnitude of this increase is prospectively associated with increased risk for anxiety and depressive disorders (Adam et al., 2014). In addition, the CAR is lower in the offspring from long-lived families (Noordam et al., 2012) and heightened levels of the CAR have been associated with impaired endothelial function in women (Violanti et al., 2009). Furthermore, the CAR has proven to be a relatively robust phenomenon and evidence suggests that it is associated with explicit measures of NA (Wüst et al., 2000; Adam et al., 2006). For example, Adam et al. (2006) observed that higher levels of NA (feelings of sadness, loneliness, and feeling overwhelmed) across the day predicted higher CAR levels the next morning. In addition, – as mentioned above – lower levels of implicit PA have already been linked to higher morning levels of cortisol (Quirin et al., 2009b). Thus, in line with previous research by Adam et al. (2006) on explicit NA, we expected that prior-day mean implicit negative memory bias and implicit NA would be significantly positively associated with the CAR the next day. In addition, we predicted a negative relationship between implicit PA and the CAR the next day, indicating that higher levels of implicit PA are associated with reductions in the CAR. Lastly, we hypothesized that implicit memory bias and implicit affect – assessed directly after awakening – were significantly associated with the CAR on the day itself.

## MATERIALS AND METHODS

### PARTICIPANTS

Participants were undergraduates from the social science faculty of Leiden University. The study was approved by the local ethics committee. Participants were told that they were participating in a study on emotions in daily life to keep the hypotheses with regard to unconscious stress/emotions unknown. Participants were rewarded with research participation credits or 20 €.

Based on the effect that Quirin et al. (2009b) found [*r*(30) = -0.46 for PA and *r*(42) = 0.40 for NA] we calculated that to achieve the same effect size in our study at least 35 participants (power = 0.80, alpha = 0.05) would be required. Fifty-six participants signed up by registering themselves in scheduled time slots online. Individuals who smoked, used drugs, used chronic medication known to affect cortisol levels, or anyone who currently had a pulmonary-, cardiovascular- or mental health problem were excluded because of its known effect on cortisol (Kirschbaum and Hellhammer, 1989). Due to these recruitment demands, we had to exclude one participant, because of smoking. Our sample consisted primarily of psychology students with a mean age of 20.8 years (SD = 2.4, Range 17–27 years) of which 19 were men and 36 women. Forty-eight participants were of Caucasian descent, four of mixed descent, one African, one Hindu, and one Arabian.

### PROCEDURE

Participants signed up for a specific time slot, starting at 9 am, 11 am, or 12.30 pm. After being given a brief overview of the study-procedures participants gave written informed consent. Thereafter, we assessed basic demographic variables as well as the exclusion criteria. The current study was part of a larger study, including a laboratory part (with several tests assessing emotion regulation; not reported here) and an ambulatory part, which required participants to wear an ECG belt (not reported here), keep an electronic diary, and to sample saliva at set times, the latter being the focus of our research. After the laboratory part participants were informed of the ambulatory procedure. They were explained how the ECG apparatus and the salivettes worked and given a manual containing instructions for cortisol sampling as well as information on the state questionnaires, favorable eating times and information related to the other parallel studies. With regard to the implicit measures (word fragment completion and IPANAT), participants were told that we wanted them to play several “language or word-games” that could best be played if they used their intuition to complete these games, and that there were no right or wrong answers.

During the study we made use of ecological momentary assessments to assess the variables of interest (salivary cortisol, implicit affect, explicit affect, and bio-behavioral variables). For 24 h, excluding any time between 10 pm and 10 am (to ensure no interference during sleep), participants were triggered by an alarm on a smartphone to answer several questions each full hour. Considering their return to the lab 24 h later this resulted in about 12 triggered alarms per person. During three of these times (11 am, 3 pm, and 9 pm), participants also had to sample saliva for later cortisol analysis. Time slots for these assessments were chosen in such a way that we could examine stress-related changes in the cortisol profile throughout the day and compare them taking their temporal relationship in consideration (Kirschbaum and Hellhammer, 1989). On top of these measurements at fixed intervals, participants were required to complete a morning assessment directly after waking up which consisted of first taking a saliva sample, then filling in the IPANAT and several questions about sleep, and finally, after 30 min, another saliva sample to measure the CAR.

### INSTRUMENTS

#### Cortisol sampling

Participants were instructed to sample their saliva by chewing on a cotton dental role, consequently absorbing the saliva. Thereafter, the saturated cotton role is placed in a plastic tube with a lid (Salivette^®^, Sarstedt, Essen, Belgium). Upon reception we stored the tubes at -20° celsius until further analysis. Importantly, we instructed participants on how to take the samples. During this we emphasized that any smoking or consumption beforehand, or touching of the dental role during the sampling would affect the results and should therefore be avoided as much as possible. The tubes were sent to the bio-psychological lab of Prof. Dr. C. Kirschbaum (TU Dresden, Dresden, Germany) where unbound (free) cortisol (in nmol/l) in saliva was determined using a commercially available chemiluminescence immunoassay (CLIA; IBL-International, Hamburg, Germany).

To obtain the CAR we simply subtracted the second morning measurement (waking + 30 min) from the baseline morning measurement (waking). Previous research has shown that this sampling procedure is sufficient to estimate the CAR (e.g., Adam et al., 2006; Chida and Steptoe, 2009) and that difference scores from waking to 30 min yield similar scores to more intrusive methods using more samples (Williams et al., 2005).

In addition, cortisol samples that were flawed (e.g., saliva not sampled within a 10 min interval after the alarm went off (for daytime cortisol) or when the second sample in the morning was not taken within 40 min after awakening) were coded using a separate variable (e.g., no compliance). Furthermore, we added a second variable indicating any food intake in the 30 min before the sample was taken.

#### Word fragment completion task

A word fragment completion task was constructed to assess implicit negative memory biases (Graf et al., 1982; Quirin et al., 2009a). At each assessment, participants were required to complete four word fragments as fast as they could. The fragments were constructed using the following rules: (1) each fragment could be completed as negative word, (2) Each word consisted of four letters, (3) each of these fragments already contained two letters (e.g., “B_O_), and (4) dictionaries listed at least four alternatives for completing each fragment to form a word of four letters, and (5) negative words were never the most common completion of the fragment.

To obtain negative bias scores, the words were finally rated by three raters (including one of the authors; BV) on whether the valence of the words was negative or not (1 or 0). To estimate interrater reliability, intraclass correlations were calculated which showed high agreement between the raters (all ICC[3,k] > 0.89). Finally, total scores were dichotomized to obtain a measure of the presence of a negative memory bias (minimally one negative word coded as ‘1’) or the absence of such a bias (‘0’).

#### Implicit affect

Unconscious stress was measured with a modified version of the IPANAT (Quirin et al., 2009a). The IPANAT originally is a paper and pencil test that asks participants to rate to what extent certain artificial or pseudo-words express certain moods. It uses a cover story telling participants that “*The following words are from an artificial language. They are intended to express various moods. In all languages, there are words that help to express their meanings by the way they sound (for example, the word ‘rattle’ almost sounds like something that rattles). In poetry and literature, this is known as onomatopoeia*.” (cited from: Quirin et al., 2009a, p. 503). Thus, participants are led to believe that the test is about finding onomatopoeias, whereas it is assumed that the ratings that they make actually express the amount of implicit affect.

The original IPANAT consists of six pseudo-words (e.g., ‘segam,’ ‘haswi’) and participants are asked to rate on a scale from one to four how much this pseudo-word is expressing a certain affect (Dutch: blij, geërgerd, energiek, gespannen, bedroefd, goedgehumeurd; English: happy, irritated, energetic, tense, sad, merry). For our ambulatory study we created an ambulatory version of the IPANAT, thereby slightly modifying it. Instead of providing the participants with all pseudo-words and all affect words at once, participant in our ambulatory study were presented one pseudo-word per hour. They subsequently rated the extent to which this pseudo-word expressed the six affective states, with one combination of a pseudo and an affective word per screen. To create a Dutch version of the IPANAT we selected 12 pseudo-words from a validated list of 30 neutral pseudo-words. Though it might have had implications for the internal consistency of the test, the reasoning behind this modification of the IPANAT is that because we administered the IPANAT each hour we believed that using a reduced version of the IPANAT would form less of an interruption in daily life, but would still provide us with accurate data to assess state implicit affect. Other than Quirin et al. (2009a) we changed the scales from a ‘1 to 4’ format to scales that ran from ‘1 (not at all)’ to ‘6 (very much)’ to add response variability in order to measure minor fluctuations. The implicit NA subscale was computed as the mean of the ratings for the negative adjectives (i.e., irritated, tense, sad) at each assessment and the implicit PA subscale as the mean for all positive adjectives (i.e., happy, energetic, merry) at each assessment.

Cronbach alphas for implicit PA and NA – for the original version of the IPANAT – are each 0.81, with 1-week test-retest reliabilities of 0.72 for implicit PA and 0.76 for implicit NA (Quirin et al., 2009a). The original IPANAT further shows adequate internal validity with primary loadings on congruent components between 0.8 and 0.9 and all cross-loadings lower than 0.10. Moreover, the positive and negative scale were uncorrelated indicating the presence of independent constructs, *r*(203) = 0.03, n.s. (Quirin et al., 2009a). Because the psychometric properties of the ambulatory IPANAT are unknown, we decided to report these properties in the results section.

#### Explicit affect

To examine explicit affect as the conscious counterpart of implicit affect we formulated four basic emotional questions that cover the width of emotions (see also Pieper et al., 2007). Participant were asked to rate if they have felt this emotion (angry/irritated, happy/joyful, let down/sad, restless/tense) during the past hour. Much like the IPANAT, these answers were also scored on a scale from 1 (not at all) to 6 (very much). Like the IPANAT we also combined the explicit affect scores into a subscale for explicit PA and a subscale for explicit NA (see above).

#### Stress and worries

Each hour, participants were asked whether they had experienced any stressful event (yes or no) or any episodes of worry (yes or no) or whether participants were anticipating a stressful event in the next couple of hours (yes or no). Yes-answers to either of these questions also led to questions on intensity and duration of the event (see also Pieper et al., 2007).

#### Bio-behavioral data

Lastly, we finished each assessment with a small behavioral questionnaire. These questions comprise bio-behavioral assessments on physical activity and intensity, whether they smoked (yes/no), and how much coffee or alcohol they consumed (NB, Although being a smoker was an exclusion criterion, it was still possible that ‘recreational smokers’ entered the study; two participants indeed indicated to have smoked one cigarette during the evening, excluding these participants did not alter the results).

#### Materials

The apparatus used for momentary assessment is the android-operated Motorola Razr Smartphone. The use of a digital device in a momentary study provides many advantages over a paper-based study (e.g., usability, portability, data transfer, and data export). The application MovisensXS (https://xs.movisens.com/) was used to trigger the forms for assessment on the phones.

### STATISTICAL ANALYSIS

Firstly, we explored whether our modified version of the IPANAT showed psychometric properties that were similar to the original one (Quirin et al., 2009a). Therefore we conducted a principal components analysis on the adjectives ratings using varimax rotation. Furthermore, we looked at the reliability of the subscales and the cross-correlations of the subscales with the other psychological variables, the latter to obtain insight into the validity of the implicit measures. With respect to the reliability, we used the approach put forward by Cranford et al. (2006) and Shrout and Lane (2012), who use a generalizability theory approach to estimating reliability coefficients. Thereafter we tested our main hypotheses.

Multilevel growth curve modeling was used to examine the relationship between implicit negative memory bias, the IPANAT subscales and cortisol levels (Singer and Willett, 2003). Because the raw cortisol data were non-normally distributed, we successfully performed a natural log transformation to meet the model’s basic assumptions. We chose multilevel analysis because it allows for data to have a hierarchical structure, and takes into account that intra-individual measurements are correlated. In this study affect measures are gathered at time points which are nested within persons. Therefore, our model contains two levels: an “episode level” with measurements that vary across the hourly assessments and a “person level” with demographic and stable person level variables (i.e., age, gender, BMI, use of oral contraceptives, and ethnicity). Episode level variables were time of day (coded as: 0 = 11 am, 1 = 3 pm, 2 = 9 pm), the implicit measures (IPANAT subscales and the implicit negative memory test), explicit affect measures, occurrence of stress-events and worry episodes, as well as the bio-behavioral variables general activity, smoking, coffee, alcohol, and food intake. Continuous episode level variables (except for time of the day) were initially grand mean centered. When using grand mean centered time-varying variables, the slopes estimated in the analyses represent a composite of both within and between subjects effects (e.g., Hoffman and Stawski, 2009; Peugh, 2010).

For the ultradian cortisol data we first assessed the bivariate associations between the predicting variables (implicit negative memory bias, implicit PA, implicit NA) and cortisol levels, while controlling for the effects of time of the day. Thereafter, we assessed the independent contribution of the variables by entering them all as predictors in an overall model. Finally, we added the explicit measures and the bio-behavioral variables to examine whether the observed effects were not explained by these latter variables. Error covariance was best modeled using the diagonal structure.

To assess the second set of hypotheses pertaining to the CAR, we aggregated the implicit memory bias and IPANAT data from the first measurement day into a prior-day variable. To examine the relation between the CAR and current and prior-day implicit measures, we used bivariate Pearson correlations.

Analyses were performed in SPSS version 20.0 (MIXED and GENLIN MIXED) and R (lme4 and qgraph packages) with a two-tailed alpha set at 0.05.

## RESULTS

Fifty-five participants were, in total, prompted to answer hourly forms 904 times, yet ignored the forms 110 times, dismissed them in 34 occasions and they were incomplete 11 times. Here we will focus on the 227 prompts that occurred simultaneously with the saliva sampling. From these 227 prompts, 35 were missed and 8 assessments were incomplete. Two participants were excluded from all analyses because they responded too fast to the questions and had too many identical responses (e.g., 1-1-1-1-1-1 responses on the IPANAT). With regards to the CAR measurements, 3 participants did not sample the CAR (i.e., forgot to take a second sample after 30 min).

As a way of ascertaining the validity of the implicit assessments with the IPANAT, during debriefing participant were asked to list what they thought we intended to measure with this test. Because our research was entitled “emotions in daily life” about half of the 55 people listed the obvious link they thought the study had with emotions. Only four participants mentioned associations with stress or unconscious emotions, excluding these participants from subsequent analyses did not significantly change the results.

Descriptive statistics for the measured variables are given in **Table 1**. In comparison with most similar ambulatory cortisol studies (van Eck et al., 1996; Smyth et al., 1998; Jacobs et al., 2007) our sample was relatively young. In line with data found by Quirin et al. (2009a; See Table 1), participants showed a bias toward both implicit PA and explicit PA (i.e., higher scores for positive than for NA). With respect to the negative memory bias, assessed with the word fragment completion task, 50% of all assessments were associated with at least one word that was completed negatively. Additionally, in 7.9% of the assessments a stressful event was reported and in 18.7% of the assessments an episode of worry was reported. Calculated alternatively, participants reported a mean of 0.21 (SD = 0.46) stress-events and a mean of 0.49 (SD = 0.78) worry episodes, which is lower compared to previous ambulatory studies by our group (e.g., Ms > 1; Verkuil et al., 2012).

**Table 1:** Descriptive statistics.

	*N*	*M*	SD	Minimum	Maximum	%
**Person level**
Gender	53					64.2% women
Age	53	20.70	2.37	17	27	
BMI	53	22.05	2.36	18.19	28.41	
Ethnicity	53					88.5% Caucasian
Contraceptive use	34					67.6% of all women
**Episode level**
Implicit PA	174	3.35	1.20	1	6	
Implicit NA	173	2.84	1.02	1	5.67	
Implicit negative memory bias	170					50%
Explicit PA	132	3.95	1.23	1	6	
Explicit NA	132	2.01	0.83	1	5	
Worry episode	139					18.7%
Stressful event	139					7.9%

**FIGURE 1:**
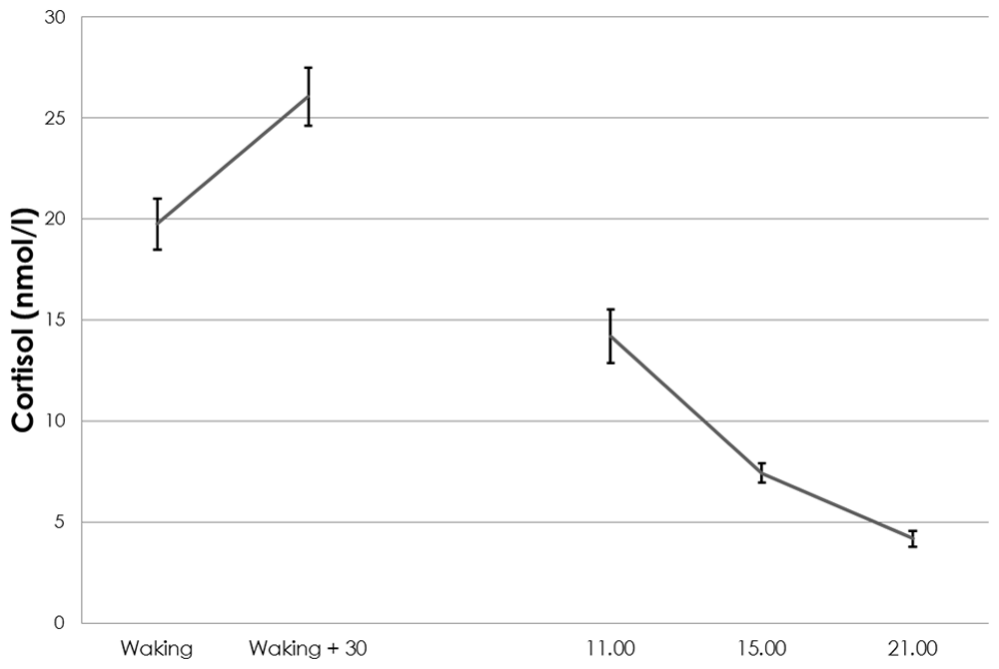
**Ultradian cortisol levels (±1 SE)**.

**Figure 1** shows that the cortisol levels followed the expected ultradian pattern, with a clear increase in cortisol directly after waking up and an overall decrease of cortisol during the day.

### PROPERTIES OF THE MODIFIED IPANAT AND ASSOCIATIONS WITH OTHER STRESS-RELATED VARIABLES

A principal components analysis using varimax rotation was conducted on the IPANAT ratings to examine whether our modified version of the IPANAT would possess a similar factor structure as the original version. Based on eigenvalues being bigger than one, two components were extracted, explaining 64% of the variance. The results from **Table 2** clearly provide evidence for the existence of the two proposed subscales: implicit PA and implicit NA, and thereby supports the construct validity of this modified IPANAT version.

**Table 2:** Varimax rotated loadings of ratings on the IPANAT items.

IPANAT item	Factor 1	Factor 2
Happy	0.845	-0.278
Energetic	0.802	0.130
Merry	0.809	-0.181
Tense	-0.08	0.841
Irritated	-0.205	0.629
Sad	-0.230	0.729

We additionally calculated the reliability of the subscales. We calculated the reliability pertaining to individual differences in the average ratings on the IPANAT subscales (aggregated across the four assessments). Results showed that between person reliability of the average ratings was satisfactory for implicit PA, *R*_kf_ = 0.84 and for implicit NA, *R*_kf_ = 0.81. That is, when averaging the ratings across all four assessments, the means are quite stable and seem to reflect individual differences.

**FIGURE 2:**
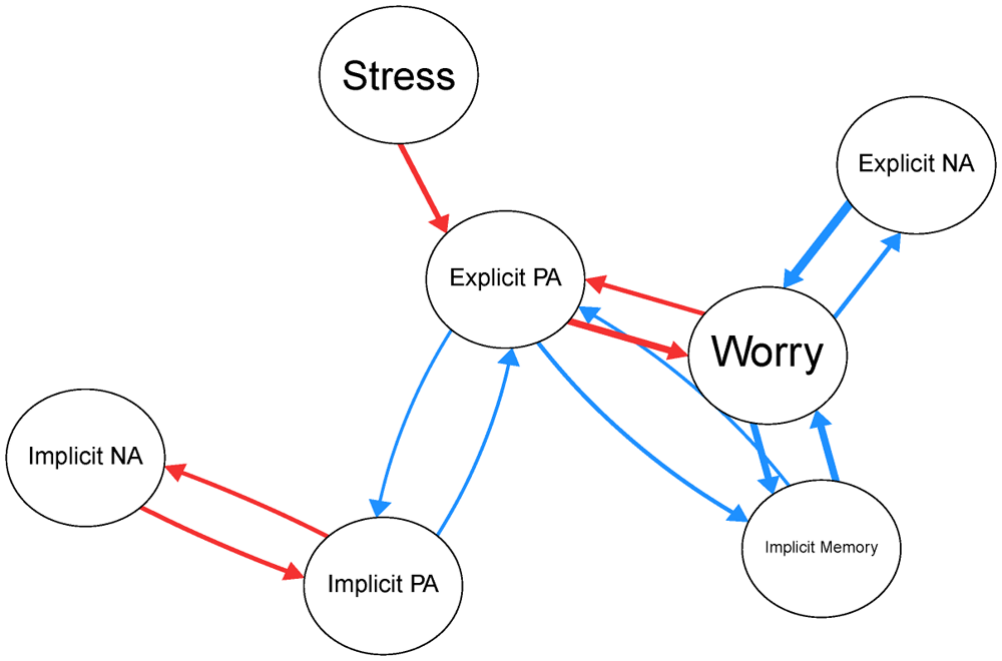
**Significant associations between the psychological variables.** Blue lines indicate positive associations and red lines indicate negative associations. Thicker lines represent stronger associations.

Multilevel analyses showed that momentary assessed implicit PA was negatively associated with implicit NA, *B* = -0.667, *p* < 0.001, 95% CI [-0.830, -0.504]. This association remained significant when controlling for explicit affect, stress, and worry. Next, we examined the association between the implicit affect and explicit affect, stress and worries. **Figure 2** shows the results of multilevel analyses wherein each psychological variable is predicted by the other variables. This figure makes clear that while controlling for the other variables in the model, there were significant associations between implicit PA and explicit PA and implicit NA and explicit NA. Additionally, worry and stress were not independently associated with implicit affect. Stressful events were associated with reduced explicit PA and worry episodes were associated with reduced explicit PA, increased explicit NA and increased implicit memory bias.

### ASSOCIATION OF IMPLICIT AFFECT WITH CORTISOL

Using natural log transformed cortisol levels assessed at 11 am (*n* = 45), 3 pm (*n* = 48), and 9 pm (*n* = 46) as our dependent variable, we found that time of the day had a significant fixed effect on cortisol: *B* = -0.548, *p* < 0.001, 95% CI [-0.637, -0.459]. This baseline model showed that during the day, cortisol levels decreased. Next, we added the three implicit measures in three separate models, which led to three better fitting models [all χ^2^(1) > 26, *p*s < 0.001] compared to the baseline model. While controlling for time, implicit negative affective memory bias was positively associated with cortisol (*B* = 0.179, *p* = 0.012). The associations between cortisol and implicit PA (*B* = -0.041, *p* = 0.161) and implicit NA (*B* = 0.002, *p* = 0.940) were not significant (tested two-tailed). Subsequently, we examined the independent associations between the implicit measures (the IPANAT subscales and implicit memory bias) and cortisol levels. When adding all three implicit measures to the baseline model, a better fit was obtained [χ^2^(3) = 57.07, *p* < 0.0001]. **Table 3** shows that cortisol levels were negatively associated with implicit PA [*B* = -0.134, 95% CI (-0.229, -0.039)], but were – unexpectedly – also negatively associated with implicit NA [*B* = -0.106, 95% CI (-0.201, -0.011)]. In addition, cortisol levels were positively associated higher levels of implicit memory bias [*B* = 0.196, 95% CI (0.060, 0.333)].

**Table 3:** Estimates of fixed effects predicting log-transformed cortisol levels.

Parameter	*B*	SE	*df*	*t*	*p*	Interpretation^†^
Intercept	2.565	0.078	84.75	32.61	0.000	12.93 nmol/l
Time	-0.621	0.056	64.54	-11.00	0.000	46% decrease per period
Implicit PA	-0.111	0.039	82.83	-2.81	0.006	10% decrease
Implicit NA	-0.098	0.044	80.16	-2.21	0.030	9% decrease
Implicit memory bias	0.196	0.068	67.13	2.87	0.005	21% increase

*^†^Cortisol values were transformed using the natural log transformation, coefficients (B) were transformed to percentage change in cortisol per unit change in the predictor, using the formula: percentage change = 100(e^B^ – 1) (cf. Adam et al., 2006).*

We additionally explored whether the associations were driven by either between subjects effects or by within subjects effects. To do so, we entered the ratings averaged per person, across the assessments (the person mean, representing between subjects effects) as well as the ratings centered on the personal mean (person mean centered, representing within subjects effects; cf. Hoffman and Stawski, 2009). Results (not reported here) showed that only between subjects effects accounted for the associations between the implicit measures and cortisol levels.

In a final step, the explicit measures (affect, stressful events, and worries) were added to the model, but this did not improve model fit [χ^2^(4) = 2.956, *p* = 0.565]. Furthermore, none of the explicit measures was significantly associated with cortisol levels. Interestingly, this was also the case when only including time of the day and the explicit measures as predictors of cortisol, showing that explicit affect measures were not related to cortisol. Inclusion of bio-behavioral variables (caffeine intake, physical activity, smoking, alcohol intake, BMI, use of contraceptives) and personal characteristics (gender, age, ethnicity) did not lead to different results.

In order to specify the association between implicit affect and cortisol, we explored the associations between cortisol and the separate implicit affect variables (merry, happy, energetic, irritated, tense, sad), while controlling for implicit negative memory bias and time of the day. Cortisol was not significantly associated with any of these specific affect variables, although a trend appeared for implicitly feeling energetic (*B* = -0.045, *p* = 0.076).

### CURRENT-DAY AND PRIOR-DAY IMPLICIT AFFECT AND THE CORTISOL AWAKENING RESPONSE (CAR)

Measured over 50 participants we found that within the first 30 min of waking, baseline cortisol increased by about 73% showing an increase of 7.00 nmol/l (SD = 9.17). Maintaining the criterion for defining a cortisol response (2.5 nmol/l increase; Wüst et al., 2000) we observed a CAR in 64% of the subjects.

#### Prior-day implicit measures and the CAR

**FIGURE 3:**
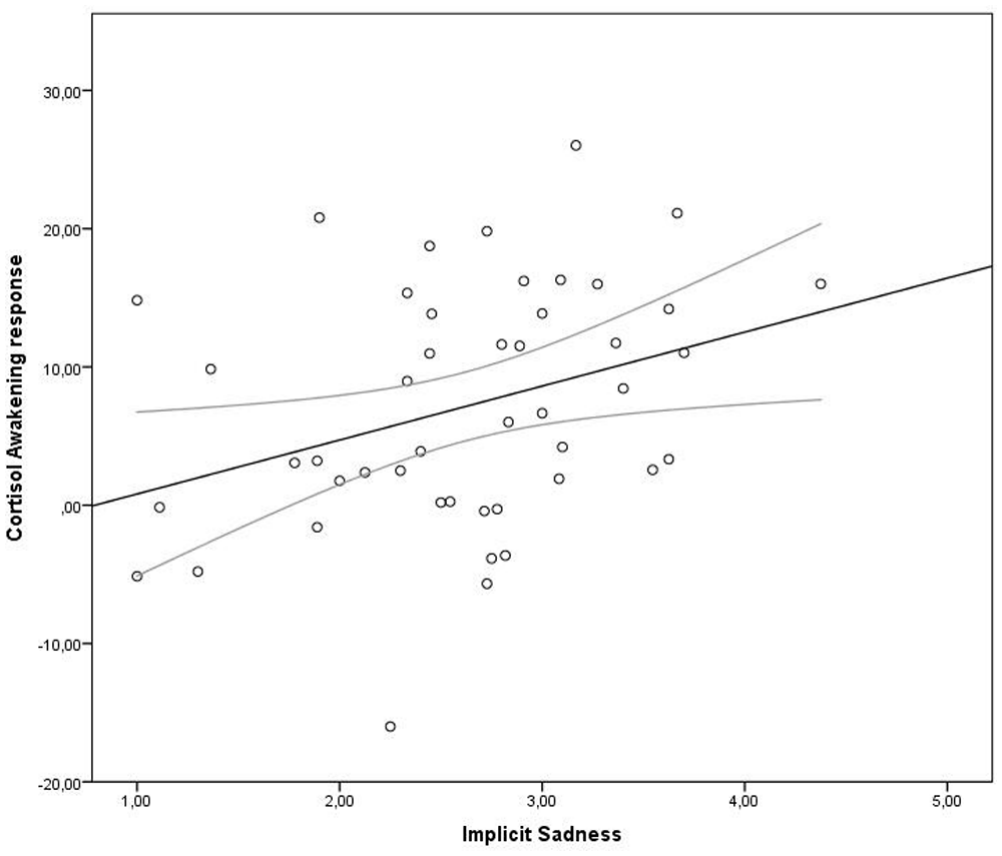
**Scatterplot of the association between prior-day implicit sadness and the cortisol awakening response (CAR)**.

Pearson correlations showed that the CAR was not related to the aggregated prior-day measures of implicit PA [*r*(48) = -0.159, *p* = 0.271], implicit NA [*r*(48) = 0.208, *p* = 0.148], or implicit memory bias [*r*(46) = 0.038, *p* = 0.797]. Inspection of whether the CAR was related to any specific prior-day affect revealed a significant association between implicit sadness and the CAR, *r*(48) = 0.291, *p* = 0.041. This association is depicted in **Figure 3**, indicating that increased levels of implicit sadness are associated with increases in the CAR. The CAR was not associated with explicit affect, stress, or worries during the previous day (*p* > 0.05).

#### Current-day implicit measures and the CAR

Nine participants missed the morning assessment of the IPANAT and 13 did not fill in the word fragment completion task. Pearson correlations showed that the CAR was not related to the morning assessments of implicit PA [*r*(39) = 0.068, *p* = 0.673], implicit NA [*r*(39) = -0.031, *p* = 0.847], or implicit memory bias [*r*(35) = -0.232, *p* = 0.167]. In addition, none of the specific implicit affect variables was associated with the CAR.

## DISCUSSION

The purpose of this study was to examine whether unconscious stress, assessed using measures of implicit affect (IPANAT) and implicit memory biases (word fragment completion task), is related to cortisol secretion in daily life. Our main finding is that implicit memory bias, implicit NA, and implicit PA were associated with cortisol levels across the day, but only when they were simultaneously used to explain the variance in cortisol levels. Furthermore, we could not replicate the findings of previous studies showing that explicit PA and NA, worries and stressful events are associated with cortisol levels (van Eck et al., 1996; Smyth et al., 1998; Sher, 2004). Surprisingly, when assessing the implicit measures separately, only the implicit memory bias proved to be significantly associated with cortisol secretion. Furthermore, in reviewing the associations with the CAR, we found a significant association with prior-day implicit sadness. Below these results will be discussed in more detail.

This study was the first to examine the relation between implicit measures of affect and cortisol levels in daily life. The results are partly in line with the hypothesis that unconscious stress can have physiological effects (Brosschot et al., 2010). That is, we found that participants with on average higher levels of implicit negative memory bias showed enhanced cortisol levels. Thus, when people completed one or more word fragments in a negative manner, they showed higher cortisol levels than people who completed all word fragments in a neutral or positive manner. Yet, of the three implicit affect variables, only implicit memory bias, as assessed with the word fragment completion task, was associated with cortisol levels. However, we did find that both IPANAT subscales (PA and NA) were associated with reduced ultradian cortisol levels but only when controlling for the implicit negative memory bias. These associations may have become significant due to suppression, which occurs when the association between an independent variable and the dependent variable becomes stronger through the addition of a third variable; in this case, when all three implicit measures are added to the model (MacKinnon et al., 2000). This would mean that the cortisol effect of implicit negative memory bias would suppress the effect of implicit affect. Such a process is difficult to understand, and is not easy to reconcile with the assumption that the two tests would measure, at least partially, the same phenomenon. Moreover, interpretations of these results have to be regarded with caution given the absence of direct relations between cortisol and the IPANAT subscales. That high implicit PA was associated with reduced cortisol levels seems consistent with Quirin et al. (2009b), who found that high trait implicit PA was associated with reduced total circadian cortisol secretion. In contrast to our expectation, we also found that implicit NA was associated with reduced ultradian cortisol levels. Furthermore, we also did not observe that state fluctuations of implicit affect or implicit negative memory biases were associated with fluctuations in cortisol levels within participants. That is, our findings demonstrate that trait differences between participants in these variables accounted for variation in cortisol. Longer assessment periods, i.e., longer than 24-h, might be more suited to capture the proposed within subject associations, which are possibly more subtle and only visible when measuring multiple days per person.

In addition, in our analysis of prior-day and current-day affect and its relationship with the CAR, we found that – in line with our expectation – prior-day implicit sadness was positively associated with the CAR. Taking all cortisol assessments together, the results provide a somewhat paradoxical picture. Implicit NA seemed negatively associated with cortisol during the day – albeit only after controlling for the other implicit measures, yet prior-day implicit sadness (a part of implicit NA) was positively associated with morning cortisol levels. First of all it has to be acknowledged that these results were obtained from exploratory analyses, without controlling for family wise error rate. Replication studies are therefore warranted to examine the robustness of the current findings. A possible – yet tentative – explanation for the current findings is that the discrepancy in the results can be explained by the fact that the CAR is considered an adaptive response that, based on the experiences of the day before, proactively secretes more or less cortisol secretion to anticipate on today’s stressors (Adam et al., 2006). Put in perspective, because momentary implicit sadness does not require an immediate action tendency in the current moment, there is no immediate need for heightened cortisol levels (Roseman et al., 1994); however, implicit sadness from the day before would increase your cortisol the next morning in order to be better prepared for stressors than you were the day before (which most likely would have caused the implicit sadness the day before).

Finally, in examining the relation between implicit PA and the CAR, we were not able to replicate the results found by Quirin et al. (2009b). Quirin et al. (2009b) found that implicit PA was related to cortisol specifically to the moments after awakening, although they did not compute a CAR. In our study, the aggregated measure of prior-day implicit PA and the momentary assessment of implicit PA in the morning were both not associated with the CAR. All in all, although we did find some associations between the IPANAT and cortisol levels, future studies are clearly warranted that address whether the IPANAT is associated with stress-related physiology.

## CONCLUSION

The study took place in a small homogenous group of largely female psychology students. Therefore the results found in this study may not be accurately generalized to other populations. In addition, the word fragment completion task that was used in the current study was devised to capture negative memory biases (all fragments could be completed using negative words). It might be worthwhile to examine positive memory biases as well, as such biases might buffer the negative effects of negative biases. Furthermore, although our ambulatory study was suited to address moment-to-moment associations between implicit processes and cortisol in daily life, the direction of these observed associations remains to be addressed in future studies. It might be worthwhile to use training procedures such as attentional bias modification to change implicit processes and examine changes in stress-related physiology in daily life. At least in one study by Dandeneau et al. (2007) it was demonstrated that reducing vigilance for threat was associated with reduced levels of cortisol (Dandeneau et al., 2007). Another limitation is that we were not able to randomize the order in which the word fragments and the IPANAT words were presented to the participants. Every participant reacted to the same stimuli (fragments/non-sense words) in the same order. This introduces the problem that, for example, no associations between cortisol and the morning assessments of the IPANAT and the word fragment completion task were observed, because the stimulus material at this assessment was less suited to capture implicit affect. Therefore, we recommend that future studies use a sampling method wherein the stimuli are presented to the participants in randomized order.

All things considered, this was the first study to examine the association between cortisol and implicit affect measures in daily life. The finding that implicit negative memory bias (assessed with a word fragment completion task) was associated with higher levels of cortisol in daily life, supports the recently formulated hypothesis that unconscious stress influences stress-related physiology (Brosschot et al., 2010). Yet, the results pertaining to the other instrument to assess implicit affect (IPANAT) were less consistent, although they suggest that implicit sadness predicts a stronger cortisol increase at awakening. This preliminary evidence that in daily life our stress physiology may be responsive to unconscious stress, opens up new venues for future studies and suggest that by focusing on explicit affect and conscious stress – we might have been studying just the tip of the iceberg.

## Conflict of Interest Statement

The authors declare that the research was conducted in the absence of any commercial or financial relationships that could be construed as a potential conflict of interest.
